# An Accelerometer Based on All Silica In-Line Fiber Fabry-Perot Etalon for High Temperature up to 800 °C

**DOI:** 10.3390/mi13040548

**Published:** 2022-03-30

**Authors:** Jiang Qian, Pinggang Jia, Qianyu Ren, Hua Liu, Li Qin, Jijun Xiong

**Affiliations:** 1 State Key Laboratory of Dynamic Measurement Technology, North University of China, Taiyuan 030051, China; qjiangnuc@163.com (J.Q.); pgjia@nuc.edu.cn (P.J.); renqianyu13115@126.com (Q.R.); liuhuanuc@163.com (H.L.); xiongjijun@nuc.edu.cn (J.X.); 2 Key Laboratory for Micro/Nano Technology and System of Liaoning Province, Dalian University of Technology, Dalian 116081, China

**Keywords:** high temperature, accelerometer, optical fiber, in-line, Fabry-Perot etalon

## Abstract

High-temperature accelerometers have been widely used in aerospace, nuclear reactors, automobile technologies, etc. In this paper, a fiber-optic Fabry–Perot accelerometer (FOFPA) with a cantilever beam for high temperature is designed and experimentally demonstrated. The FOFPA is formed by bonding an all-silica in-line fiber Fabry–Perot etalon (ILFFPE) to one surface of the uniform cantilever beam with the lumped mass at the free end for acceleration measurement. The all silica in-line fiber FP etalon is made by welding two gold-coat single-mode fiber (GSMF) and a hollow silica glass tube (HST). The research results indicate that the sensitivity of the FOFPA is 0.02328rad/g, and the resonance frequency is 1146.6 Hz in the range of 1 g ~ 10 g. The high-temperature performance of the FOFPA was also evaluated. From 20 °C to 800 °C, the temperature drift is about 0.3178 nm/°C. The FOFPA has the potential of being applicable in higher temperatures compared to conventional accelerometers.

## 1. Introduction

In the past twenty years, high-temperature accelerometers have played an important role in aerospace, energy exploration, gas pipeline transportation, nuclear reactor development, and the automotive industry [1,2,3,4,5,6]. For example, thermoelastic vibration testing techniques, which are conducted for supporting flutter analysis of subsonic and supersonic vehicles at high temperatures, are limited by the maximum operating temperature of the accelerometer [7]. In a nuclear power plant, components or the fuel cladding at high temperatures in the reactor need to be monitored in real-time in order to ensure the safety and the functionality of the plant structures [8,9].

In recent years, many types of high-temperature accelerometers have been reported, such as piezoelectric sensors, piezoresistive sensors, capacitive sensors, and optical fiber sensors. Jiang et al. [10] proposed a piezoelectric vibration sensor, which used a Ba2TiSi2O8 (BTS) single crystal as the piezoelectric material, for structural health monitoring at elevated temperatures (650 °C). Eklund et al. proposed a MEMS high-temperature piezoresistive vibration sensor based on SOI, which can work at 350 °C [11]. Utz et al. presented a high precision MEMS-based capacitive accelerometer, which was assessed at temperatures between −40 °C and 120 °C [12]. Shi et al. proposed an accelerometer based on the strain and bend characteristics of long-period fiber grating (LPFG). The LPFG can withstand temperatures up to 800 °C [13]. Chen et al. presented an in-line fiber-optic Fabry–Perot Sensor that can work at 500 °C [3]. Compared with these traditional electrical sensors, fiber-optic sensors have the advantages of immunity to electromagnetic interference, and optical interference is not influenced by temperature and has been widely studied [14,15,16,17,18].

In this paper, we present a FOFPA with the structure of all silica ILFFPE bonding on one surface of the constant cross-section silica cantilever with a lumped mass was bonding at the free end of the cantilever. The ILFFPE was constructed by welding an HST at the end face of two GSMFs. The structure of the FOFPA was designed using theoretical calculation and numerical simulation. The lumped mass and the constant cross-section silica cantilever work as a spring-mass damping system and the variation of the surface of the cantilever affects the length cavity of the ILFFPE. Moreover, a dual-wavelength demodulation system was used to demodulate the output of the FOFPA, which worked from 20 °C to 800 °C. The experimental results indicated that the FOFPA exhibited a low thermal drift and good nonlinear. Because the silica cantilever and the ILFFPE can withstand high temperatures, the proposed FOFPA has the prospect of measuring vibration at high temperatures.

## 2. Fabrication and Principle

**Figure 1:**
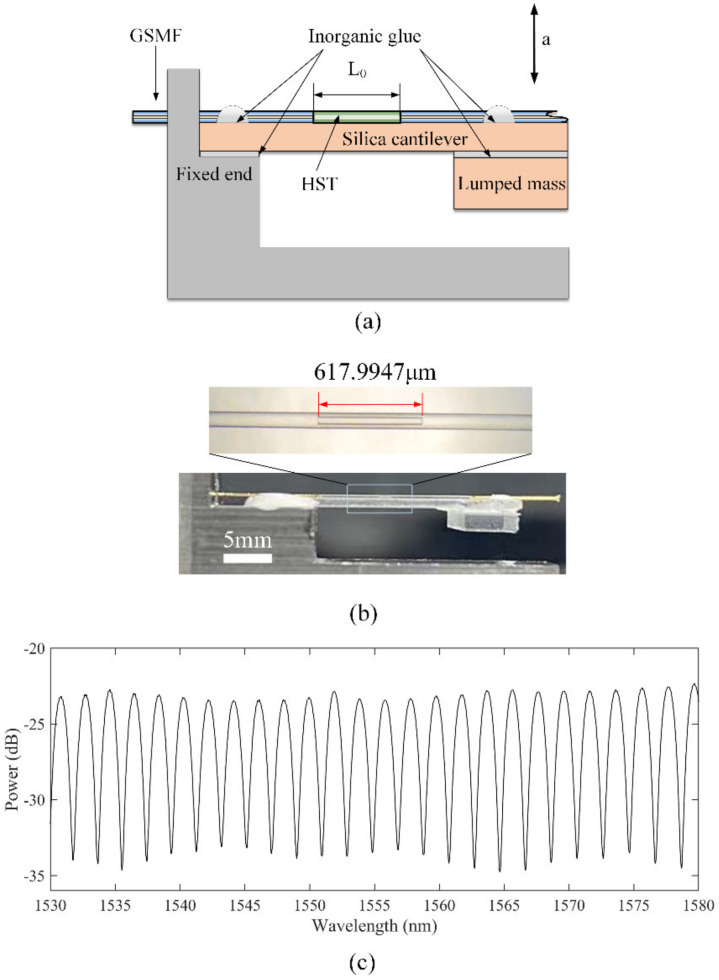
Schematic of the FOFPA based on all silica ILFFPE. (**a**) schematic of the FOFPA. (**b**) photograph. (**c**) spectrum of the FOFPA.

Figure 1 shows the schematic and photograph of the FOFPA. The FOFPA consists of an ILFFPE, a cantilever, and a lumped mass. All of them are made of silica which has a low CET. The ILFFPE is formed of a GSMF with the end face of R1, an HST with an outer diameter of 125 μm and an inner diameter of 50 μm, and another GSMF with the end face of R2, as shown in Figure 1a. The silica lumped (length: 5 mm, width: 5 mm, thickness: 1 mm) mass is bonding at the free end of the silica cantilever (length: 15 mm, width: 5 mm, thickness: 0.3 mm). The fixed end of the cantilever is bonded to a stainless steel base by inorganic glue, as shown in Figure 1b. The silica cantilever with a lumped mass at the free end is used as a mass-spring-damper system and translates the inertia force of the mass into the length change of the ILFFPE, and the initial length of the FP is 617.9947 μm.

When on-axis acceleration excitation is applied to the FOFPA, ignoring the mass of the cantilever, the strain of the silica cantilever with a lumped mass at the free end can be expressed as

(1) $$\varepsilon_{c}=\frac{m(l-x)ha}{2EI}$$

where m is the equivalent mass of the inertial block. h is the thickness of the cantilever, E is the Young’s modulus of silica, l is the length from clamped end to free end of the cantilever. a is the acceleration applied on the inertial mass. x is the position on the cantilever from the clamped end. I is the moment of inertia of the cantilever, and expressed as $I=bh^{3}/12$,where b is the width of the cantilever.

According to Equation (1), the strain of the silica cantilever is dependent on the position of the silica cantilever. The initial length cavity variation of the IEFFPA can be expressed as

(2) $$\Delta L_{\mathrm{FP}}=\int_{l_{0}}^{l_{0}+L_{0}}\frac{m\eta (l-x)ha}{2EI}dx=\frac{m\eta ha}{2EI}(lL_{0}-\frac{1}{2}L_{0}^{2}-l_{0}L_{0})$$

where $l_{0}$ is the position from clamped end of the cantilever, $L_{0}$ is the initial FP length of the ILFFPE, η (≤1) is the strain transfer coefficient which subscribes the relationship between the silica cantilever and ILFFPE.

Therefore, the phase shift of the ILFFPE output from the FOFPA generated by acceleration excitation can be expressed by

(3) $$\Delta \phi =\frac{4\pi n\Delta L}{\lambda }=\frac{24\pi nm\eta }{\lambda Ebh^{2}}(lL_{0}-\frac{1}{2}L_{0}^{2}-l_{0}L_{0})a$$

where n is the refractive index of the air in the FP cavity and n≈1. λ is the wavelength of laser. According to Equation (3), if the structure and sizes of each part of the FOFPA are finished processing, the Δφ is linear with a.

According to conservation of energy, the resonance frequency of the silica cantilever is given by

(4) $$f_{res}=\frac{1}{2\pi }\sqrt{\frac{Ebh^{3}}{4l^{3}(m+\frac{33}{140}m_{c})}}$$

where $m_{c}$ is the mass of the cantilever beam.

**Figure 2:**
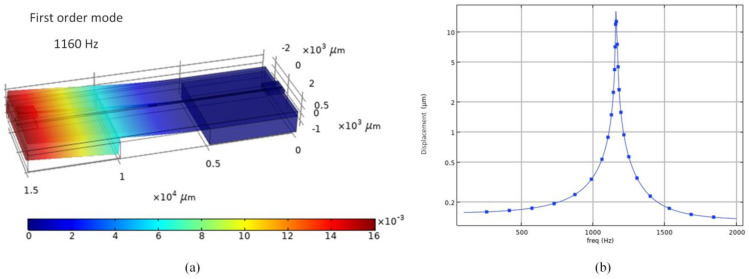
The finite element simulation results of FOFPA. (**a**) the first order mode of FOFPA. (**b**) frequency domain analysis of FOFPA.

The finite element simulation of the structure is carried out, and the simulation results of the resonance frequency of FOFPA is obtained, as shown in Figure 2. It can be seen that the frequency point corresponding to the maximum deformation of FOFPA from 100 Hz to 2000 Hz is 1111 Hz, which is consistent with the calculation result of formula 4 in Table 1.

The FOFPA consisted of an ILFFPE, a silica cantilever, and a silica mass. The ILFFPE was made by two GSMFs (ASI9/125/155G, Fiberguide Industries, Ltd., New Jersey, NJ, USA) and an HST (YN350450, Yongnian Ruipu Chromatogram Equipment Co., Ltd., Yongnian, China). The HST has an inner diameter of 65 µm and an outer diameter of 125 µm, and its outer diameter matches the optical fiber. The cantilever beam, which has a length of 15 mm, a width of 5 mm, and a height of 0.3 mm, was cut from a two-inch silica wafer with a height of 0.3 mm. By the same method, the mass with a length of 5 mm, a width of 5 mm, and a height of 1 mm was also cut from a two-inch silica wafer with a height of 1 mm.

The manufacturing process of FOFPA includes the fabrication of the ILFFPE, the bonding of the cantilever beam and mass, and the bonding of the package shell. GSMF and HST were cut flat with a cutter (CT30, Fujikura, Ltd., Tokyo, Japan) and placed into a welding splicer (62S, Fujikura, Ltd., Tokyo, Japan) for discharge welding, and the discharge parameters are 5 bit (about 4.3 W) and 200 ms. Before the welding, a length of approximately 5 mm of gold coating from the welding end face was stripped by a blade in order to weld HST. Then the HST welded to the GSMF was cut again by the cutter, and the cut of the HST was welded with another GSMF. The discharge parameters are exactly the same as the first welding. Finally, the ILFFPE was bonded with the cantilever beam by an inorganic glue (YK–8927, Yikun glue, Macheng, China), and the mass cut from the silica wafer was also bonded with the cantilever beam by the same method. Figure 1b shows the photograph of the ILFFPE and the FOFPA. As shown in Figure 1c, the Spectral contrast of FOFPA is about 10 dB. The detailed parameters of the F-P vibration sensor are listed in Table 1.

## 3. Results

### 3.1. Testing System

**Figure 3:**
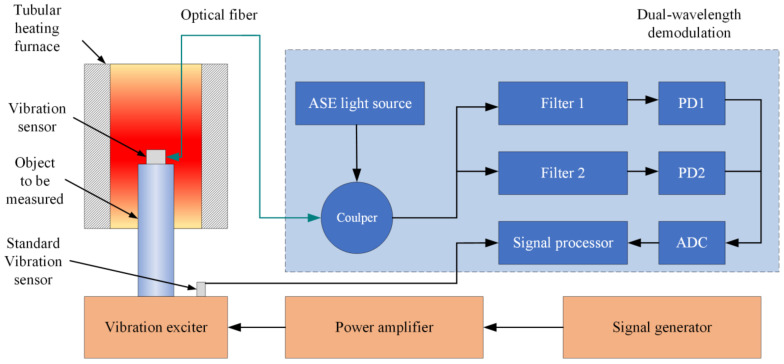
The experimental system set up of FOFPA for high-temperature testing.

Figure 3 shows the schematic diagram of the sensor test system. It consisted of a vibration excitation system, a tubular heating furnace, a standard vibration sensor, and a dual-wavelength demodulation system. The FOFPA and a metal rod are connected a by thread, and together put into the tubular heating furnace (GSL–1100X–S, HF Kejing, Hefei, China). The metal rod was connected to the vibration exciter (TV 50101, Tira, Thuringen, Germany) by a thread. The light derived from the ASE light source reached the FOFPA through the single-mode fiber and the 3 dB optical fiber coupler. The coupler split the beam into two paths that passed through two optical fiber broadband filters with center wavelengths of 1548.14 nm and 1552.744 nm. The bandwidth of both filters was 0.8 nm. Two interferometric signals at each center wavelength were obtained using two photodiodes (PDs), PD1 and PD2 (New Focus, 2503-FC-M). The detectable wavelength of the PDs ranges from 900 to 1700 nm. The voltage signals were collected by analog-to-digital conversion (ADC) and transmitted to a signal processor. More detail about the dual-wavelength demodulation system available in references [19].

### 3.2. Experimental Results

**Figure 4:**
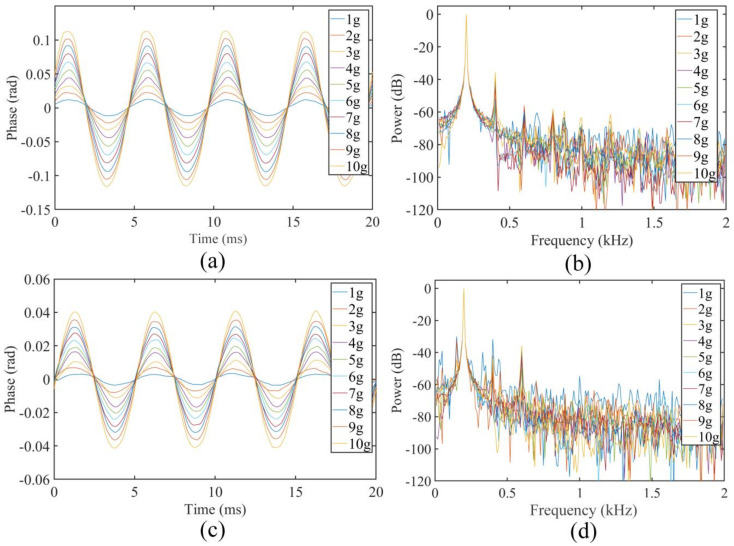
Output of the sensor from 1 g to 10 g under 200 Hz at 20 °C and 800 °C: (**a**) waveform of the sensor at 20 °C, (**b**) frequency response of the waveform at 20 °C, (**c**) waveform of the sensor at 800 °C. (**d**) frequency response of the waveform at 800 °C.

The FOFPA was evaluated under temperatures ranging from 20 °C to 800 °C, in increments of 100 °C. At each temperature step, the sensor was tested from approximately 1 g to 10 g, in increments of 1 g. The excitation frequency was 200 Hz. When loading the vibration, the accelerations were kept constant for 1 min at each point to record the vibration output accurately. Figure 4a,c shows the vibration waveform of the sensor at 20 °C and 800 °C, respectively. Figure 4b,d indicates that the sensor had the same frequency response at 200 Hz.

**Figure 5:**
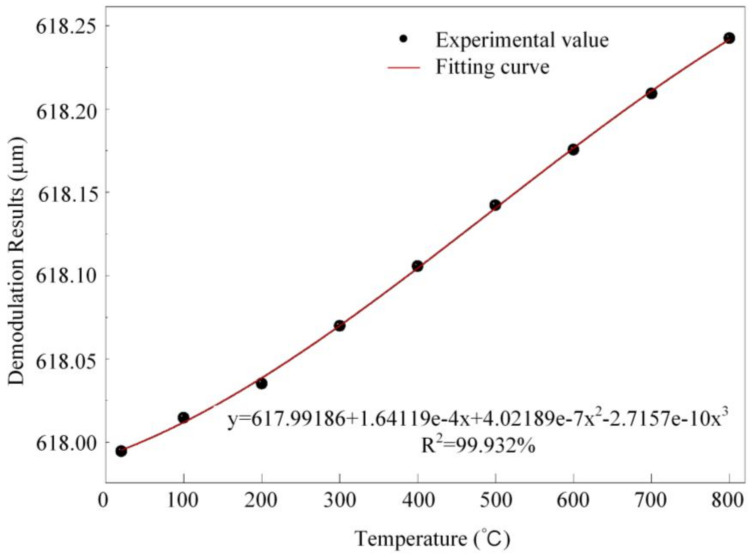
Temperature drift of FOFPA from 20 °C to 800 °C.

The temperature drift of the FOFPA was obtained by recording the interference spectrums at each temperature using a spectrometer. Then we used the method proposed in reference [20] to calculate the length cavity of the FOFPA at each temperature. The results are shown in Figure 5. According to Figure 4, the cavity length at different temperatures was cubically fitted, and the R-square is 0.99932. The length cavity of ILFFPE at 800 °C is 618.2426 μm, while the length at 20 °C is 617.9947 μm. Therefore, the temperature drift of the FOFPA is approximately 0.3178 nm/°C.

**Figure 6:**
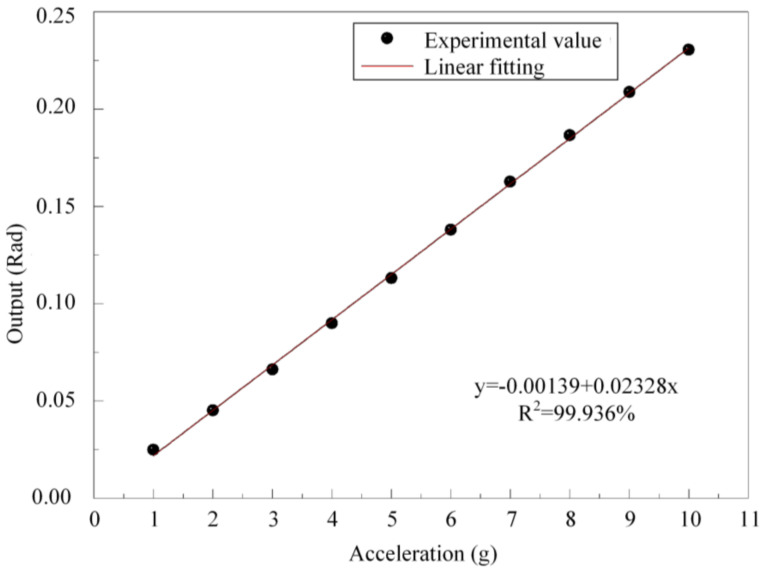
Linear fitting of the FOFPA.

We recorded the peak-peak output of the sensor at each acceleration under 20 °C. Figure 6 illustrates the linearity of the FOFPA at 200 Hz, 20 °C. The FOFPA exhibits an acceleration sensitivity of 0.02328 rad/g with an R-square value of up to 0.99936.

**Figure 7:**
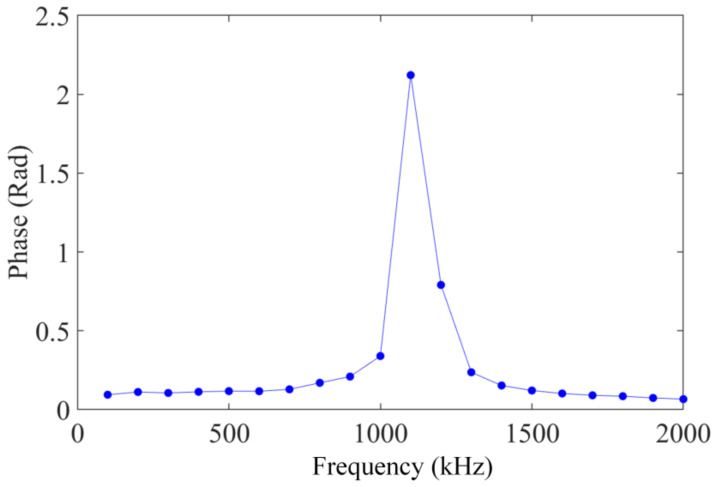
The relationship between excitation frequency and the output of the FOFPA.

The FOFPA was evaluated at 5 g under frequencies ranging from 100 Hz to 2000 Hz, with increments of 100 Hz, at 20 °C. The variation relationship of the phase output with frequency is shown in Figure 7. The output phase of the FOFPA had no obvious change in the range of 100~700 Hz, which indicated that the resonance frequency of the FOFPA was approximately 1100 Hz. This was consistent with the resonance frequency (1146.6 Hz) calculated theoretically in Table 1.

## 4. Discussion

When the acceleration and frequency remain unchanged, the output of the FOFPA at 800 °C is lower than the value at 20 °C because Young’s modulus of the silica decreases with the increase in temperature. This phenomenon shows that the acceleration sensitivity of the FOFPA decreases with the increase in temperature. However, the temperature drift of the FOFPA increases with the increase in temperature because of the thermal expansion of the structure. The residual stress caused by the thermal expansion of silica may be lower when each part of the FOFPA is made of the same material. In addition, the relationship between the acceleration and the output-phase peak is linear, which is in agreement with the prediction in Equation (3). Furthermore, the resonance frequency is mainly dependent on the shape and the size of the cantilever, according to Figure 6 and the value in Table 1 calculated by Equation (4). This means the frequency measurement range of the FOFPA can be changed in order to meet the requirement of certain specific areas, and the resonance frequency can be predicted by Equation (4).

At present, there are many commercial accelerometers that can be used in high-temperature environments. The maximum working temperature of the CA250M8XX piezo accelerometer from Vibro-Meter is 777 °C. The maximum operating temperature of Endeveo’s 6240M10 piezo accelerometers is 649 °C. The 712E/CC712E piezoresistive accelerometers can work under 260 °C. The GYD104 piezo accelerometer from Shanghai Silicate Research Institute can work under 482 °C. The ADXL206 dual-axis MEMS accelerometer developed by Analog Devices supports an ambient temperature range of −40 °C to +175 °C. The proposed FOFPA is experimental but proved to work at 800 °C. Therefore, the proposed FOFPA has the potential of being applicable at high temperatures.

## 5. Conclusions

In this paper, a FOFPA with a cantilever beam for high temperature was designed and experimentally demonstrated. The FOFPA is formed by bonding an ILFFPE to one surface of the uniform cantilever beam with the lumped mass at the free end for acceleration measurement. The experimental results show that the sensitivity, resonance frequency, and the measured range of the FOFPA were 0.02328 rad/g (20 °C), 1146.6 Hz, 1~10 g, respectively. Furthermore, the sensor can work at a high temperature up to 800 °C and shows the 0.3178 nm/°C temperature drift in the form of the ILLFPE cavity length shifting, indicating its wide and bright application prospect at high temperatures.

## Figures and Tables

**Table 1:** Parameters of the FOFPA.

Parameters	Symbol	Units	Value
Length of beam	$l_{0}$	mm	9
Width of beam	b	mm	5
Thickness of beam	h	mm	0.3
Density of monocrystalline silica	ρ	kg/m^3^	2203
Length of mass	$l_{m}$	mm	5
Width of mass	$b_{m}$	mm	5
Thickness of mass	$h_{m}$	mm	1
Initial length of FP cavity	$L_{0}$	mm	0.618
Young’s modulus of silica	E	GPa	69.6
Sensitivity	S	rad/g	0.02
Resonance frequency	$f_{res}$	Hz	1146.6
